# Excited-State Proton Transfer in 8-Azapurines I: A Kinetic Analysis of 8-Azaxanthine Fluorescence

**DOI:** 10.3390/molecules25122740

**Published:** 2020-06-12

**Authors:** Jacek Wierzchowski, Bogdan Smyk

**Affiliations:** Department of Physics and Biophysics, University of Warmia and Mazury in Olsztyn, Oczapowskiego 4, 10-719 Olsztyn, Poland; jacek.wie@uwm.edu.pl

**Keywords:** fluorescent nucleobase analogs, 8-azapurines, excited-state proton transfer, time-resolved fluorescence spectroscopy

## Abstract

A super-continuum white laser with a half-pulse width of ~75 ps was used to observe the kinetics of a postulated excited-state proton transfer in 8-azaxanthine and its 8-methyl derivative. Both compounds exhibited dual emissions in weakly acidified alcoholic media, but only one band was present in aqueous solutions, exhibiting an abnormal Stokes shift (>12,000 cm^−1^). It was shown that long-wavelength emissions were delayed relative to the excitation pulse within alcoholic media. The rise time was calculated to be 0.4–0.5 ns in both methanol and deuterated methanol. This is equal to the main component of the fluorescence decay in the short-wavelength band (340 nm). Time-resolved emission spectra (TRES) indicated a two-state photo-transformation model in both compounds. Global analysis of the time dependence revealed three exponential components in each compound, one of which had an identical rise-time, with the second attributed to a long-wavelength band decay (6.4 ns for aza-xanthine and 8.3 ns for its 8-methyl derivative). The origin of the third, intermediate decay time (1.41 ns for aza-xanthine and 0.87 ns for 8-methyl-azaxanthine) is uncertain, but decay-associated spectra (DAS) containing both bands suggest the participation of a contact ion pair. These results confirm the model of phototautomerism proposed earlier, but the question of the anomalous isotope effect remains unsolved.

## 1. Introduction

**Figure 1:**
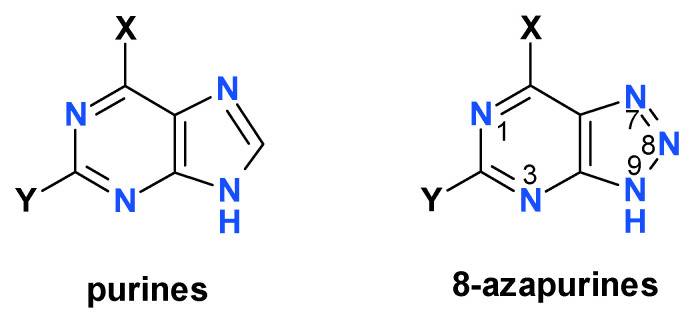
Structure of 8-azapuriners, compared to natural purines. Only one tautomeric form is shown for simplicity. Note that purine numbering is applied.

Fluorescent nucleobase analogs are often utilized to probe the structures and functions of nucleic acids and their related enzymes [1,2]. 8-Azapurines (IUPAC name: 1,2,3-triazlo-[4,5-b]pyrimidines, see Figure 1) function as fluorescent and isomorphic analogs of natural purine bases, often replacing them in numerous enzymatic processes; they are utilized, inter alia, as fluorescent probes in enzymology [3,4,5]. They are also quite toxic, but some of them reveal promising pharmacological activities, particularly against parasitic diseases [4,6]. The fluorescence of 8-azapurines and their ribosides can be applied to the quantification of enzyme activities in biological material [5,7,8], the study of polynucleotide interactions [9,10], and the study of enzyme/ribozyme structures and/or mechanisms [11,12,13,14,15].

**Figure 2:**
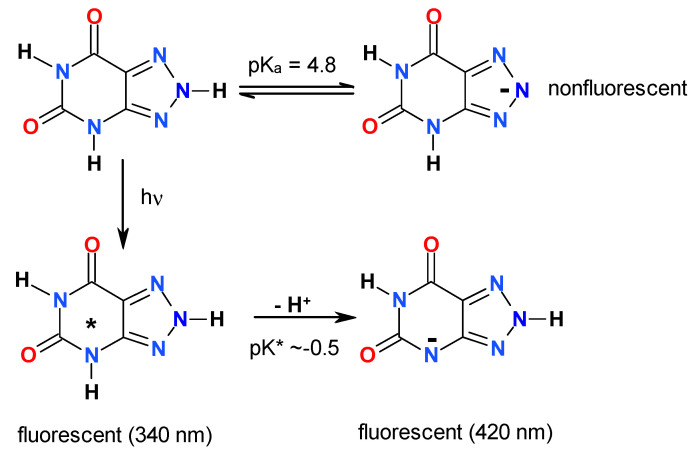
Postulated excited-state proton transfer as a source for the dual emission of 8-azaxanthine in water; adopted from [17]. The pK* for the N(3)H proton was calculated from the Foerster cycle, as applied to 8-methyl derivative (see the next section).

Some 8-azapurines, particularly 8-azaxanthine (8-azaX, see Figure 2), 8-azaisoguanine (8-azaisoG), and 2,6-diamino-8-azapurine (8-azaDaPu), exhibit solvent- and isotope-dependent dual emissions [16,17,18,19], and have been postulated to undergo excited-state proton transfer (ESPT) in protic solvents, resulting in phototautomerism [16]. The excited-state proton transfer is a well-known and widely studied phenomenon [20,21,22,23], and has resulted in the application of phototautomerism in many substituted heterocycles, like hydroxy-quinolines [21]. To date, however, only a few examples of the use of phototautomerism in nucleoside and nucleobase analogs are known [16].

The process of ESPT usually competes with other channels of the excited-state decay, leading to dual emissions; in some cases, however, the proton transfer is so rapid that ESPT is complete and as a result only one band is visible. This is particularly true for the class of “super photo-acids”, characterized by pK* < 0, including cyano-naphthols and N-substituted hydroxy-quinolines [22,23]. 

In our previous works [17,24] we analyzed the steady-state fluorescence of 8-azaxanthine and several N-methyl derivatives, particularly those modelling the dominant tautomeric structures. Here, we present a kinetic analysis of the dual emission observed in 8-azaxanthine and 8-methyl-8-azaxanthine in methanol and deuterated methanol, performed with a time resolution of ~75 ps, with the objective of verifying the proposed scheme of phototautomerism (Figure 2, below). As can be seen in Figure 2, the low pK* (−0.5) for the postulated process of ESPT in 8-azaxanthine classifies the latter as a “super photo-acid”.

## 2. Results

### 2.1. Steady-State Fluorescence of 8-Azaxanthine and Its Derivatives

**Figure 3:**
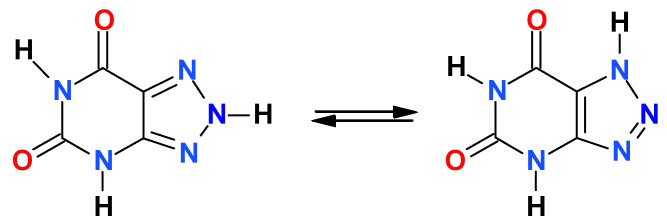
Structure of 8-azaxanthine. The compound is shown in its two most stable tautomeric forms.

**Figure 4:**
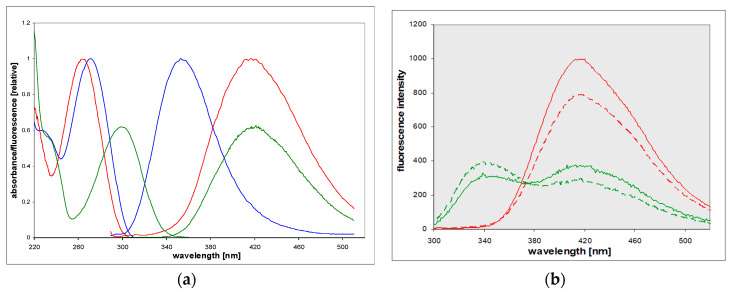
(**a**) UV absorption and fluorescence spectra of the neutral forms of 8-azaxanthine (red) and 8-azatheophylline (1,3-dimethyl-8-azaxanthine, blue) in acidified aqueous solutions, compared to the anionic form of N8-methyl-8-azaXan (green); (**b**) Emission spectra of neutral 8-azaxanthine observed in water (red), deuterium oxide (red, dashed), 99% aqueous methanol (green), and 99% deuterated methanol (green, dashed). Excitation was at 280 nm. Spectra are normalized to equal absorbance at 280 nm. Each medium contained ca. 3 mM HCl or DCl in order to assure the neutral form of the ground state.

8-Azaxanthine (8-azaXan, IUPAC name: (1,2,3-triazolo[4,5-d]pyrimidine-4,6-dione, see Figure 3) is a highly fluorescent analog of xanthine [17]. In the ground state, it is a mixture of N(8)H and N(7)H protomers [17]. Its fluorescence in a weakly acidified aqueous medium (where the neutral species is dominant) was characterized by a single band with maximum wavelength of 420 nm, which exhibited an anomalous Stokes shift of ca. 14,000 cm^−1^ [17]. 8-azaXan did not emit at pH > 6 because it underwent ground-state deprotonation from the triazole ring (pK_a_ 4.8, [3,5]), and this anion is nonfluorescent. When N(3) nitrogen was blocked, like in the N(3),N(1)-dimethyl-8-azaxanthine (8-azateophylline), its maximum fluorescence was shifted to ~340 nm (Figure 4a), but it also disappeared at pH > 5 due to deprotonation of the ground state. By contrast, methylation of N(8) resulted in the appearance of intense fluorescence with a maximum 420 nm observed at pH 2–11 [17].

In non-aqueous protic solvents such as alcohols, dual emission of 8-azaxanthine was observed (see Figure 4b and [17]), with maxima at 340 and 420 nm. The short-wavelength band, with a maximum at ~340 nm, resembled that observed in 8-azatheophylline (Figure 4a), and the 420 nm band that of the N8-methyl derivative (see the following paragraph). This dual fluorescence was also somewhat isotope-dependent, as shown in Figure 4b. The replacement of methanol by deuteromethanol (MeOD) caused an increase in the short-wavelength band and a decrease in the phototautomeric band.

**Figure 5:**
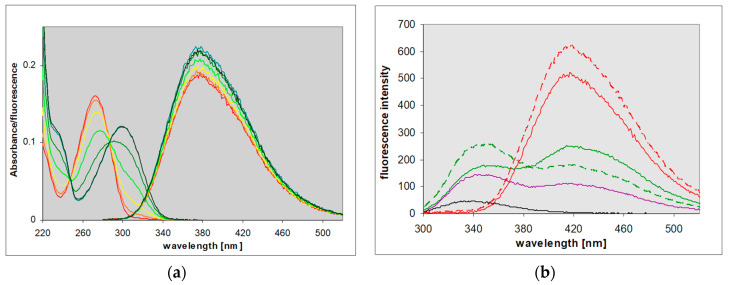
Spectra of 8-methyl-8-azaxanthine: (**a**) spectrophotometric and fluorometric titration of fluorescence excitation at 285 nm; calculated ground-state pK_a_ ~ 7.3. (**b**) Emission spectra of the neutral form in various conditions: H_2_O (red), D_2_O (red, dashed), methanol (green), deuterated methanol (green, dashed), isopropanol (violet), and dioxane (black); spectra in water and D_2_O are divided by 2. Each solvent contained 1% aqueous acetic acid (0.2 M). Spectra were excited at 280 nm and normalized to equal absorbance at 280 nm.

8-Methyl-8-azaxanthine is a model of the main tautomeric form of 8-azaxanthine [17]. Its fluorescence in the aqueous solution was quite similar to that of 8-azaxanthine, but more intense (λ_max_ at 420 nm, yield 0.5 to 0.6), and was observed in a broad pH range (2–11). Excitation spectra were in line with electronic absorption, the latter being highly pH-dependent, reflecting the ground-state deprotonation from the N(3) nitrogen (pK_a_ ~7.05). As a result, the observed Stokes’ shift changed from ca. 12,000 cm^−1^ at pH < 6 to <7000 cm^−1^ at elevated pH (see Figure 5a). In non-aqueous protic solvents like methanol and isopropanol, an isotope-dependent dual emission was evident, but in dioxane only the 340 nm band was visible (see Figure 5b). We interpret these facts as evidence of an excited-state proton transfer [17].

### 2.2. Time-Resolved Spectroscopy of 8-Azaxanthine and Its N8-Methyl Derivative

**Figure 6:**
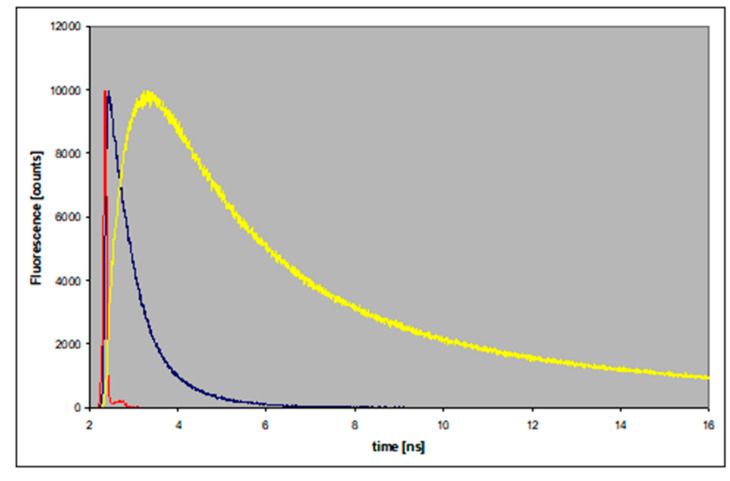
Fluorescence rise and decay (linear scale) of the neutral 8-azaxanthine in methanol/2 mM HCl, observed at 340 nm (blue) and 480 nm (yellow). Excitation was at 280 nm. The instrument response function (IRF) is given in red.

To check the above interpretation, we measured the time-resolved spectra of 8-azaxanthine and its N8-methyl derivative in methanol and deuterated methanol; that is, in the conditions where the dual emission was observed. As shown in Figure 6, the long-wavelength band (420 nm) of 8-azaXan fluorescence was delayed relative to the high-energy band (340 nm), suggesting some form of photochemical transformation. This phenomenon was not observed in aqueous media, where only the long-wavelength band was present (see the previous section).

**Figure 7:**
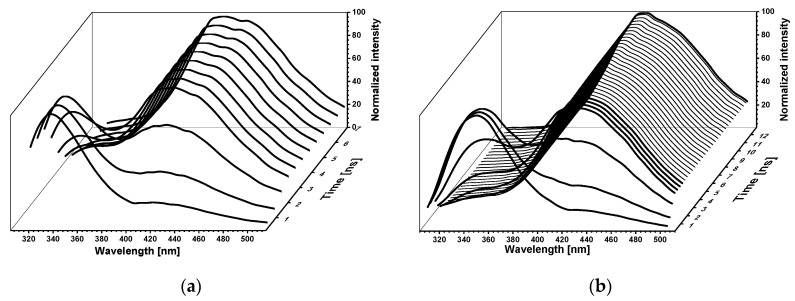
Normalized time-resolved emission spectra (TRES) of 8-azaxanthine (**a**) and its 8-methyl derivative (**b**). Spectra were calculated with a 0.4 ns step. Calculation range: (**a**)—0 ÷ 7 ns, (**b**)—0 ÷ 13 ns. Excitation was at 280 nm.

Normalized time-resolved emission spectra (TRES) [25] of 8-azaxanthine in 1% aqueous methanol, acidified with 3 mM HCl, are shown in Figure 7a, and those of 8-methyl-8-azaXan in Figure 7b. These spectra show different time evolutions of the two observed emission bands, again suggesting photo-transformation.

**Figure 8:**
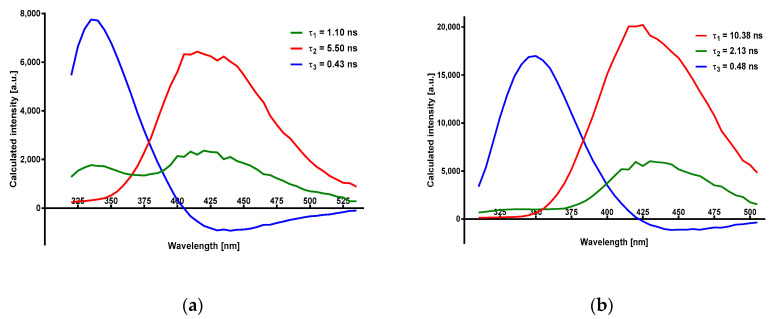
Amplitudes of the observed decay times as function of the observation wavelength calculated for (**a**) 8-azxaxanthine and (**b**) N8-methyl-8-azaxanthine in 99% aqueous methanol. Excitation was at 280 nm.

Global analysis of the observed emission decays in methanol allowed for the identification of three decay times, with results summarized in Table 1. Details of 2- and 3-exponential fits are given in the Supplementary Materials (Figures S1 and S2). The amplitudes for the individual decay components, as a function of the observation wavelength (DAS, see ref. [26]), are given in Figure 8. One of the amplitudes assumes negative values at λ > 400 nm and is interpreted as a rise-time [26].

In aqueous media (H_2_O or D_2_O), only a single-emission band was observed, and the observed fluorescence decays were monoexponential (Table 1). The observed yields and fluorescence decay times in aqueous media were ca. threefold longer than those measured in 99% methanolic solutions (see Table 1). For the model N8-methyl-8-azaxanthine, decay times as long as 14 ns were observed in D_2_O (Table 1), with a fluorescence yield reaching 60% at pH > 8 [17]. These decay times and yields were nearly constant at pH 4–10 (data not shown), and diminished at the extreme pH due to dynamic quenching [17].

## 3. Discussion

### 3.1. The Origins of Dual Emission and Calculations of pK*

In a previous paper [17], we interpreted the steady-state emission of 8-azaxanthine as the result of phototautomerism of the anionic species, as shown in Figure 2 (see the Introduction). The phototautomeric species was generated by deprotonation of N(3), instead of N(8), as observed in the ground state [3]. This interpretation was based mainly on its comparison with the 8-methyl derivative, which is incapable of deprotonating N(8)H at pH 4.8, instead losing its N(3)H proton in the ground state [3,5]. The photo-deprotonation of the N(1)H, although conceivable, is less energetically favorable. This is evident from the spectral properties of dimethyl 8-azaxanthine derivatives, reported long ago by Nubel and Pfleiderer [27]; in particular, from a comparison between the *anionic forms* of N1,N8-dimethyl and N3,N8-dimethyl derivatives, exhibiting long-wavelength maxima at 300 and 282 nm, respectively, and similar values of acidic pK in the ground state [27]. This leads to the conclusion that the former derivative must be a much stronger acid in the excited state than the latter.

Based on the Förster cycle [22], it is possible to estimate the pK* of the N8-methyl derivative. Assuming that the 340 nm emission band observed in methanol arises due to the neutral species of the molecule, and that the 420 nm band comes from its anion, a value of ca. −0.5 is obtained for the pK* [17]. Although this estimation is only semi-quantitative [22], it allows for the classification of both 8-azaxanthine and its 8-methyl derivative as “super-photoacids”, along with cyano-naphthols and N-methylated hydroxy-quinolines [21,23].

Estimation of the pK* value of 8-azaxanthine remains a more subtle question. This compound can adopt many protomeric forms and can deprotonate from several (3 or 4) acidity centers, but no thermodynamic equilibrium can be reached in the excited state, so estimation of the “true” or thermodynamic pK* is impossible. There is no evidence for ESPT in 8-azatheophylline (1,3-dimethyl-8-azaxanthine). Therefore, an analogous process of photo-deprotonation of N(3)H is postulated to occur in the 8-azaxanthine molecule, with similar pK*. This process can be observed only at pH < 5, where 8-azaxanthine exists as a neutral species in the ground state [17]. Low solubility of 8-azaxanthine in non-polar organic solvents precludes further investigations of solvent effects on the emission spectra.

Based on Weller’s relation [20], the estimated N(3)H deprotonation time in water is ca. ~10 ps [17]. This value explains why there is no visible 340 nm emission band in the acidified water and D_2_O. In alcoholic media, known to be weaker proton acceptors than water (thus slowing down the proton transfer), two emission bands were present (Figure 4b and Figure 5b). In non-polar and non-protic dioxane, the long-wavelength band disappeared. This is also typical of other super-photoacids [22].

### 3.2. Time-Resolved Spectra

Figure 6 presents a general view of the time-evolution of the spectra, showing evidence of the delayed appearance of the long-wavelength band. This kind of dependence is typical for excited-state proton transfer phenomena, resembling the best-known example of 2-naphthol [22,26].

The TRES spectra presented in Figure 7 are typical of a two-state excited-state reaction which competes with fluorescence emission [26]. This reaction runs to the product state in which energy is lower than the postulated proton donor energy level. The calculated lifetime of the product state is longer than that of the initial state (Table 1) and is connected with long-wavelength emission bands.

The decay-associated spectra (DAS; Figure 8) show amplitudes of the decay components as a function of wavelength, with one of the amplitudes running into negative values, also confirming the two-state model [26]. This is a major component (and the shortest component) of the 340 nm band decay, in agreement with the postulated mechanism. Much more difficult to interpret was the presence of three instead of two decay times, as obtained using Global Method software (see Table 1 and Figure 8). While for the parent 8-azaxanthine this fact could possibly be explained by N(8)-N(7) tautomerism of the ground state [3,5], this explanation does not work for the N8-methyl derivative.

It is now generally accepted that the process of excited-state proton transfer involves at least two separate steps; that is, initial generation of an ion pair and the subsequent diffusional process of ion separation, according to the general scheme (adopted from [22]):

(1) $$\mathrm{RNH}↔[{\mathrm{RN}}^{-}\cdots H^{+}]↔{\mathrm{RN}}^{-}+H^{+}$$

For cyano-naphthols in water, the first step (generation of the ion-pair) was characterized by rate constants ~2–7 × 10^10^ s^−1^, or rise-times ~15–50 ps [28], but this was an environment-sensitive process, so the observed rise-time of ca. 500 ps in methanol can be ascribed to this step. The long decay time of ca. 8 ns was characteristic of the free anion, so the intermediate third process, of a decay time of 1–2 ns, and the emission spectra (DAS) similar to the free anion (see Figure 8b), must represent an additional channel for the ion de-excitation. One possible explanation is ion recombination [22,29] that is at least partially reversible, as suggested by the presence of the 340 nm band in the DAS spectrum (better visible in the unsubstituted 8-azaxanthine, Figure 8a), but other processes (e.g., cage effects or geometric rearrangements) are also possible. This problem therefore requires further study.

### 3.3. Isotope Effects

The kinetic isotope effects (KIEs) of the excited-state proton-transfer reactions are typically high, ranging from 1.5 to 5 [30,31]. The observed rise-times (Figure 6 and Table 1) were not particularly dependent on isotope substitution (Table 1), suggesting that the postulated proton transfer was not the rate-limiting step in the delayed appearance of the 420 nm band, at least in the methanolic solution. At present, we are not able to explain this anomaly, but can only speculate that the first step in the process (generating an ion pair in situ), in the case of a super-acid, must be rapid (possibly ~10 ps in water [29]), and as such does not contribute to the overall KIEs. The following diffusional step (proton diffusion) is also isotope-sensitive, but the KIEs are not as prominent, and are difficult to estimate for a complex medium like aqueous methanol. We conclude that the observed low KIE values are not contradictory to the previously proposed model of phototautomerism (Figure 2).

### 3.4. Extension to Other Azapurine Derivatives

Other 8-azapurines, like 8-azaisoguanine [19] and 8-aza-2,6-diaminopurine [18], have been shown to undergo analogous excited-state proton transfer reactions, leading to phototautomerism. In contrast to 8-azaxanthine, the phototautomerism in the above-mentioned compounds can be observed for the molecules protonated in the ground state. These are cases of a “phototautomerism via the cation” [16], where the protonated species, presumably a photo-super acid, rapidly deprotonates after the excitation, producing a non-typical protomer in the excited (S_1_) state. Like in 8-azaxanthine, these processes are nearly 100% effective in acidified aqueous solutions, but in acidified alcoholic media dual emissions can be observed and rise times of the long-wavelength bands measured. Additionally, in 8-azaisoguanine and its N8-methyl derivative, a dual emission can be observed in neutral aqueous media [19], ascribed to N(1)H-N(3)H phototautomerism, catalyzed by water or buffer ions. The detailed kinetic analysis of these will be presented in a separate paper.

### 3.5. Perspectives

8-Azaxanthine derivatives are known to be biologically active, although less toxic than the analogous 8-azaguanine and 8-azaadenine derivatives [4,5]. They are good agonists or antagonists of adenosine receptors [4,32], and inhibitors of urate oxidase [4,33]. The parent 8-azaxanthine was reported to inhibit the growth of some pathogenic bacteria, including *Pseudomonas aeruginosa* [34]. The strong fluorescence of 8-azaxanthines makes them promising probes for studying receptor-binding mechanisms, enzyme–ligand interactions, and the quantification of enzyme activities [4,5]. The presented results indicate that a full understanding of this fluorescence, as well as of the somewhat similar fluorescence of other 8-azapurines [18,19], will require the application of more sophisticated experimental approaches and possibly quantum-mechanical calculation methods in order to elucidate the possible participation of conformational changes and hydrogen transfer, known to play an important role in the process of excited-state deactivation in some heterocyclics [35].

## 4. Materials and Methods

8-Azaxanthine was acquired from Sigma-Aldrich (St. Louis, MO, USA), and the 8-methyl-8-azaxanthine derivative was synthesized previously [17]. Their purity was confirmed via HPLC analysis. 1,3-Dimethyl-8-azaxanthine (8-azatheophylline) was synthesized according to known procedures from the methylated diamino-uracil derivative [24].

Steady-state emission spectra were measured on a Varian Eclipse instrument (Varian Corp., Palo Alto, CA, USA), and UV absorption kinetic experiments were performed on a Cary 5000 (Varian) thermostated spectrophotometer. Fluorescence yields were determined relative to tryptophan (0.15) or 1,N^6^-ethenoadenosine in water (0.56). Spectra, including TRES, were measured in semi-micro cuvettes, path length 4 mm, to diminish the inner-filter effect.

For TRES determination, concentrations of ca. 15 μM were used. All the spectra were corrected for inner-filter effects I and II according to the procedure described by Kasparek and Smyk [36].

Emission decays were measured using a PicoQuant FluoTime 200 spectrometer with an MCP PMT detector. Sources of excitation were measured using the SuperK EXTREME EXW-20 (High Power Systems) with a DeepUV unit (265–345 nm, 3–30 μW) from NKT Photonics (Birkerod, Denmark). A cell holder with right-angle geometry and 4 ps resolution was used. The count rate per second at the detector was kept below 1% of the laser replication rate to avoid pulse-pileup. The half-peak excitation pulse time was ca. 75 ps. Data were analyzed with Easy Tau version 2 software (PicoQuant, Berlin, Germany), using the multiexponential intensity decay model (1) as follows:

(2) $$I(t)=\int_{-\infty }^{t}\left[IRF(t^{\prime }\right)\sum_{i=1}^{n}\alpha_{i}\exp(-\frac{\left(t-t^{\prime }\right)}{\tau_{i}})]dt^{\prime }$$

where *IRF*(*t*′) is the instrument response function at time *t*′, *α_i_* is the amplitude of the decay of the *i*-th component at time *t*, and *τ_i_* is the lifetime of the *i*-th component. TRES spectra were calculated using Global Method (software provided by PicoQuant) and theory formulated by Maroncelli and Fleming [25]. Goodness of fit was estimated by calculating χ^2^ and the autocorrelation function.

## 5. Conclusions

The kinetics of the dual emission of 8-azaxanthine and its N8-methyl derivative in aqueous and alcoholic solutions strongly indicate a two-state model of photo-transformation, thus confirming the model of phototautomerism presented in Figure 2, indicating the photo-deprotonation of N(3)H as a source of the dual emission. Both 8-azaxanthine and its N8-methyl derivative can be classified as “super-photoacids”, with pK* < 0. The measured rate of proton transfer was ~500 ps in alcohols, but in aqueous media it was below the resolution limits of the used apparatus (75 ps). The kinetic analysis reveals three decay times, indicating two distinct mechanisms of phototautomer de-excitation, with time constants in methanol of ca. 8 and 1.5 ns (the exact nature of which remains unknown). In the alcoholic media this process was not very sensitive to deuterium exchange, which is not easy to explain in the present state of this investigation.

## Figures and Tables

**Table 1:** Rise and decay times of the fluorescence of the investigated 8-azapurine derivatives in 1% aqueous methanol. Each sample was acidified by 3 mM HCl/DCl. Calculation was done using Global Method, with the range of wavelength observation indicated below, in 5 nm steps.

Compound/Medium	λ_obs_ (nm) ^a^	Rise Time (ns)	Decay: τ_1_ (ns)	Decay: τ_2_ (ns)	χ^2^ (Global)
8-azaXan/H_2_O ^a^	350 ÷ 530	-	5.11 ± 0.05	-	1.110
8-azaXan/D_2_O ^a^	350 ÷ 530	-	8.076 ± 0.003	-	1.145
8-azaXan/MeOH	320 ÷ 530	0.426 ± 0.004	5.50 ± 0.13	1.102 ± 0.026	1.167
8-azaXan/MeOD	295 ÷ 500	0.510 ± 0.001	6.36 ± 0.04	1.407 ± 0.004	1.133
8-methyl-8azaXan/H_2_O	350 ÷ 520	-	11.494 ± 0.004	-	1.104
8-methyl-8azaXan/D_2_O	350 ÷ 520	-	14.55 ± 0.06	-	1.167
8-methyl-8azaXan/MeOH	310 ÷ 505	0.484 ± 0.002	10.38 ± 0.08	2.134 ± 0.037	1.155
8-methyl-8azaXan/MeOD	320 ÷ 505	0.498 ± 0.002	8.32 ± 0.02	0.865 ± 0.009	1.139
8-methyl-8azaXan/iprOH	350 ÷ 500	0.454 ± 0.004	9.60 ± 0.30	1.401 ± 0.025	1.303
8-methyl-8azaXan/dioxane	310 ÷ 370	-	0.13 ± 0.02 ^b^	-	1.279

^a^ Range of observation wavelength in 5 nm steps, with excitation at 280 nm; ^b^ This is the main component (80%); minor components are 0.45 ns (10%) and 5.4 ns (10%); no rise time was observed.

## Supplementary Materials

The following are available online, Figure S1a,b, showing best fits of the emission decay curves.
