# Supplementary material for: Excited-State Proton Transfer in 8-Azapurines I: A Kinetic Analysis of 8-Azaxanthine Fluorescence
Source: Molecules. 2020 Jun 12;25(12):2740. doi: 10.3390/molecules25122740 (PMC7356501; doi:10.3390/molecules25122740)
Supplement: Supplementary file 1 [file molecules-25-02740-s001.pdf]

Supplementary materials

Below we present analyses of 8-azaxanthine fluorescence decay in acidified MeOH with 280 nm excitation and 340 nm observation wavelength, fitted for 2 and 3 exponential components.

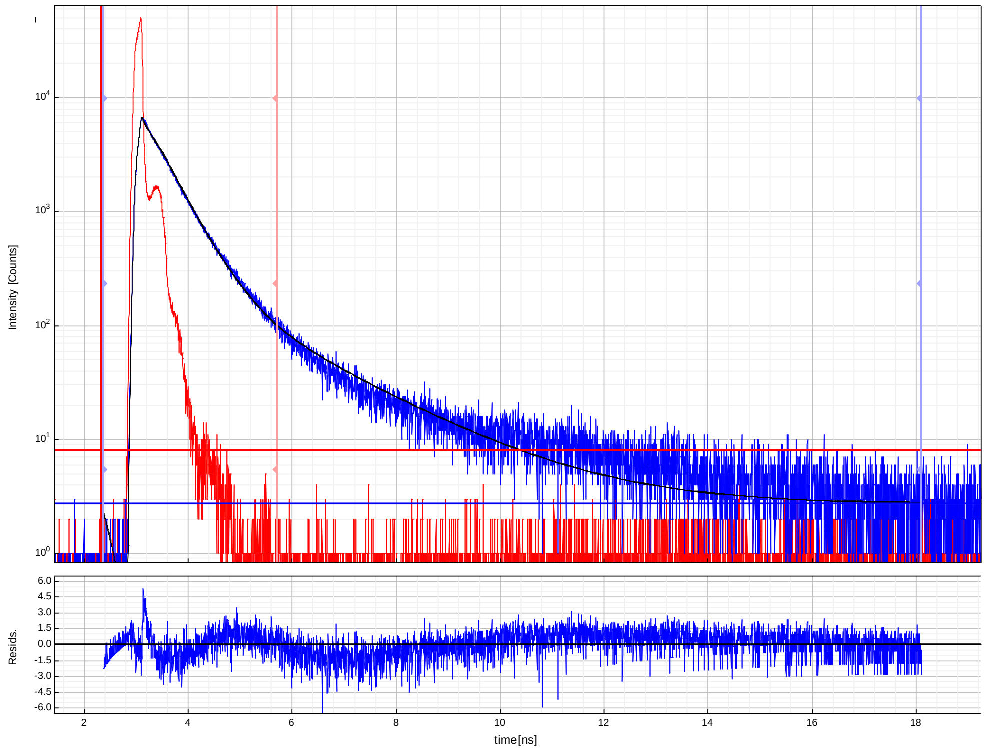

Fig. S1. Two exponential reconvolution of 8-azaxanthine, $\chi 2 = 1.657$.

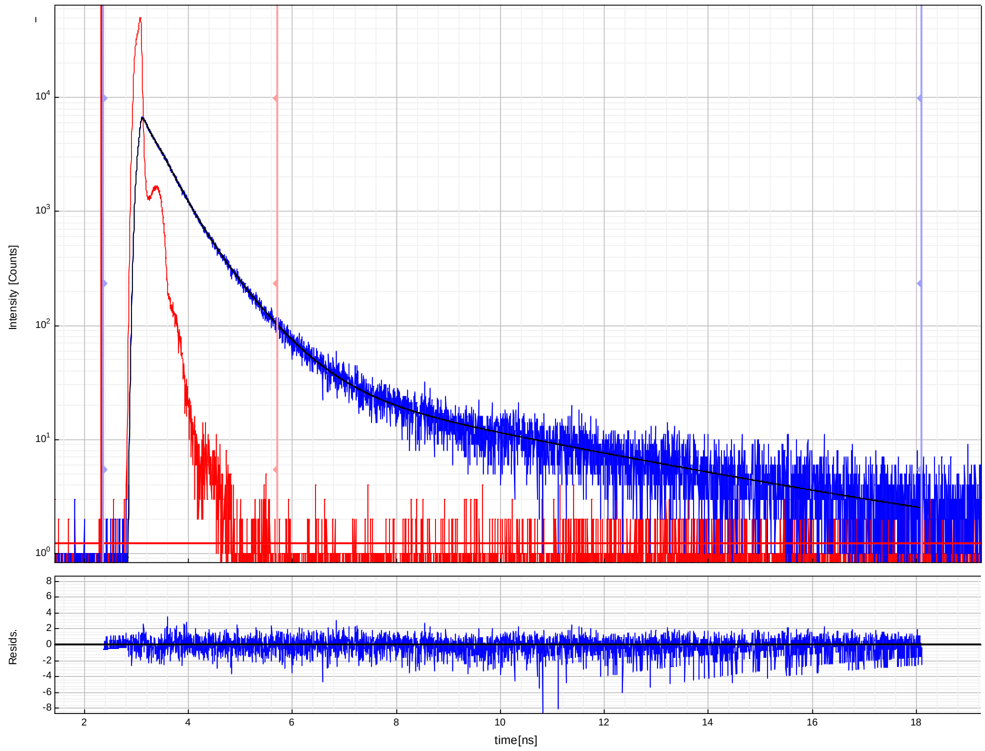

Fig. S2. Three exponential reconvolution of 8-azaxanthine, $\chi 2 = 1.237$.
